# Understanding Older Adults’ Experiences With a Digital Health Platform in General Practice: Qualitative Interview Study

**DOI:** 10.2196/59168

**Published:** 2024-08-30

**Authors:** Hanna R Knotnerus, Hà T N Ngo, Otto R Maarsingh, Vincent A van Vugt

**Affiliations:** 1 Department of General Practice, Amsterdam UMC, Location Vrije Universiteit Amsterdam, De Boelelaan 1117, Amsterdam, 1081 HV, Netherlands, 31 613275226; 2 Amsterdam Public Health Research Institute, Amsterdam, Netherlands

**Keywords:** digital health care, digital health platform, general practice, qualitative research, older adults, primary care, mobile phone

## Abstract

**Background:**

In our aging population, primary care is under pressure to remain accessible to all. Effective use of digital health care could potentially lower general practitioners’ (GPs) workload. Some general practices are already implementing a digital health platform as a primary method to contact their patients. However, it is unknown how older people experience this novel way to communicate with their GP.

**Objective:**

The aim of this study was to study the experiences of patients aged 65 years and older in general practices who use digital health as a primary communication tool. The secondary aims were to identify barriers and facilitators for the use of digital health care and whether a practice focus on digital health influences older patients’ choice to enlist.

**Methods:**

We invited all patients aged 65 years and older at 2 general practices in Amsterdam that work with a novel digital health platform. We used purposive sampling to select a heterogeneous group of patients in terms of age, sex, level of education, digital literacy, and experiences with the digital app of their general practice. We conducted 18 semistructured interviews from May through July 2023. All interviews were audio-recorded, transcribed, coded, and thematically analyzed.

**Results:**

We generated three themes: (1) experiences of older people with digital health care in general practice, (2) impact of individual factors on digital health experiences, and (3) reasons for choosing a digitally oriented general practice. Participants reported both positive and negative experiences. The main perceived advantages of the digital health platform were increased accessibility, direct GP contact without an intermediary, and saving time through asynchronous communication. The disadvantages mentioned were log-in difficulties and problems with the automated explanatory questionnaire. Individual factors such as age, digital literacy, and expectations of general practice care seemed to impact people’s experiences and could act as barriers or facilitators for using digital health. Reasons for older patients to enlist at a general practice were mainly practical. The digital orientation of the practice hardly played a role in this choice.

**Conclusions:**

Older patients in general practice see benefits to using a digital health platform that offers 2-way chat-based communication between the patient and GP. We found that individual factors such as skills, norms and values, attitudes toward digitalization, and expectations of general practice care impacted older patients’ experiences with digital health care. For many older participants, the digital profile of the general practice did not play a role in their choice to enlist. Further improvement of digital health platforms will be necessary to ensure digital health for all in general practice.

## Introduction

Primary care is under pressure. The population in Europe is aging, and the prevalence of multimorbidity rises, which will increase the demand on primary care services [1-3]. These developments will be difficult to address by the current capacity of health care professionals. Compared with 2015, British general practitioners (GPs) in 2023 already had to care for 19% more patients [4]. In a Dutch representative survey among GPs in 2022, a total of 82% of participants reported that the current workload was too high [5]. Digital health care is often mentioned as a possible solution to meet increased demand and reduce high workload [6,7].

Digital health can be defined as “the proper use of technology to improve the health and wellbeing of people, as well as enhancing the care of patients, through intelligent processing of data” [8,9]. In 2022, the majority of Dutch general practices already used digital platforms for written e-consults and reorders of medication, with similar use in urban and rural areas [10,11]. Digital health has the potential to improve both efficiency and quality of health care. However, there are risks and challenges in using digital health through these platforms. The main concern is digital inequity since digital health is less accessible for people with lower digital skills, lower (health) literacy, and lower financial status [7,8,10,12-17]. By transforming primary care without regard for digital inequity, we risk further increasing health inequity in society [8,12,13,18]. Older people, aged 65 years and older, relatively often require general practice care while they are less likely to use the internet [19,20]. Issues such as lower digital device skills, difficulties evaluating the quality of information on the internet, and concerns about privacy have been reported as barriers for older people to use digital health [10,13,21]. In designing new digital health platforms for primary care, it is therefore vital to understand and address the difficulties older patients face.

In Swedish primary care, digital health platforms that allow 2-way (asynchronous) chat-based communication between the patient and GP, (synchronous) video communication, and self-registration of patient data using automated questions have been researched in recent years [22-26]. Although proper implementation represents many challenges, these digital health platforms improve accessibility across time and space and are seen as a useful addition to current practice both by patients and GPs [22-26]. However, these studies have not specifically focused on the experiences of older adults with this novel way of communication between patients and GPs.

Recently, a few general practices in the Netherlands have started using a similar digital health platform that offers 2-way chat-based communication. To our knowledge, no scientific studies have been conducted to assess this novel form of digital health in the Netherlands. Our main aim in this study was to investigate the experiences of older patients with this digital health platform. Secondary aims were assessing barriers and facilitators for the use of the app and investigating if a practice focus on digital health influences older patients’ choice to enlist.

## Methods

### Study Design

A qualitative study was performed using semistructured interviews to get an in-depth understanding of the experiences of older people with a novel digital health platform in Dutch general practice. We followed the COREQ (Consolidated Criteria for Recording Qualitative Research) to conduct this study (Checklist 1).

### Setting

We performed this study in 2 general practices in Amsterdam, Doccs Slotervaart and Doccs Amstel III. As is common in Dutch general practice, enlisting in these practices as a patient is only possible for people who live in the same postal code area. Both practices have worked with a digital health platform (doccs app; doccs BV) since their start in 2021. The doccs app allows 2-way (asynchronous) chat-based communication between the patient and GP and is designated to be the primary way for patients to contact the GPs. The app is solely a communication tool in which patients can chat with their GP, make appointments, or order repeat medications. The app does not have an automated role in the diagnostic or treatment process and is not linked with the electronic patient record. The app has a 2-factor authentication log-in process and is secured with end-to-end encryption to comply with the General Data Protection Regulations. The app can be used on a smartphone or tablet. The patient starts a chat conversation with the GP through the app when they want to make an appointment or ask a question. The patient is automatically asked several explanatory questions about the complaint (shown in Multimedia Appendix 1) that help the GP provide an answer to the patient’s question or prepare for a face-to-face consultation. It is possible for patients to skip these questions. Within practice hours, one of the GPs of the practice will review and provide a direct answer within 1 hour. To ensure accessibility for all, it is made clear to patients that use of the app is not required to enlist as a patient because appointments can also be made by telephone or by visiting the practice.

### Recruitment of Participants

At the time of recruitment, the total number of patients in the practices was 5055. We approached all patients aged 65 years and older in both practices (N=163) by postal mail. We sent the first invitation on May 19, 2023, and a reminder letter on June 8, 2023. Both letters contained an invitation, study information, and an informed consent form. In total, 54 (33%) patients responded that they wanted to participate. We called all respondents and used purposive sampling to select a group that was heterogeneous in age, sex, level of education, digital literacy, and experiences with the digital health platform. The level of education was assessed according to the Dutch Statistical Office [27]. Perceived digital literacy was assessed by using the eHealth Literacy Scale (eHEALS) questionnaire, which is validated for use in older Dutch adults [28-30]. All respondents provided written informed consent. Within reflexive thematic analysis, the term data saturation is deemed less useful since there are always new theoretical insights possible when data continue to be collected [31]. Regarding the breadth of our research questions and pragmatic considerations, we hypothesized that we would need to conduct 10‐20 interviews. The final sample size was determined in the process of data collection and reviewing data quality. Recruitment continued until the research team agreed that the data set contained enough richness and complexity to address the research questions.

### Interviews

All interviews were performed in Dutch from May through July 2023. We compiled the topic list based on discussions within the research team and previous research [8,10]. During the interviews, the interviewer constantly revisited the topic list and added items when relevant (final topic list in Multimedia Appendix 2). Most interviews took place at the patient’s home or the general practice, 1 interview took place at the Amsterdam University Medical Centers. The interviews lasted 22 to 66 minutes. The lead author (HRK) performed all interviews. She explained to patients beforehand that she was not linked to the doccs general practice and guaranteed patients that their audiotape would be deleted after transcription. All interviews were audiotaped and transcribed verbatim.

### Analysis

We used a reflexive thematic analysis approach according to Braun and Clarke [32-34]. After reading the transcripts several times, the data were coded by 2 authors (HRK and HTNN) with an inductive orientation. At the time, HRK was a medicine master student in her final year of training, and HTNN was a general practice resident and PhD student. HRK had an open view toward digital health for older adults in general practice but was mindful of the dangers it may pose to the accessibility of care, especially for older patients. HTNN shared those concerns, but as a PhD candidate who researched the implementation of a web-based treatment for chronic dizziness in general practice, she also had a lot of positive experiences with older patients using digital health. HRK and HTNN separately coded the first 2 transcripts and then reflected together on the story within the data. After their discussion, HRK continued coding the other transcripts to further analyze the data. Next, HRK assigned codes to generate initial themes and subthemes which were visualized in a mind map. This preliminary analysis was discussed in a meeting with HRK, HTNN, and VAvV. Afterward, HRK continued analyzing the data, also performing selective coding, in which she conceptualized each theme further, searched for relations across cases, and analyzed variation within and between the cases. She visualized this in mind maps, code matrices, and code-relation matrices to gain insight into the spectrum of different factors influencing patients’ experiences. The final results were discussed with the project team (HRK, VAvV, and ORM). Interviews were analyzed in Dutch using MAXQDA (version 2022; Verbi Software). After completion, all themes, subthemes, codes, and quotes were translated into English by the authors.

### Ethical Considerations

The study received institutional research board approval by the medical ethics committee of the VU University Medical Center (2023.0001). The study was conducted in accordance with the ethical standards of the responsible committee. All participants included in the study provided written informed consent. Data were deidentified after collection and handled in accordance with the Amsterdam Public Health research institute code of conduct, following General Data Protection Regulation rules. Participants received no financial compensation to participate, but travel costs, when applicable, were reimbursed.

## Results

### Overview

We conducted and analyzed a total of 18 interviews with older patients before the research team agreed the data set was rich and complex enough to answer the research questions. Characteristics of participants are described in Table 1.

**Table 1.** Participant characteristics.

Participant	Sex	Age (years)	Digital literacy^a^	Level of education^b^
A	Male	69	29	High
B	Female	68	39	High
C	Male	77	31	High
D	Female	69	40	High
E	Female	70	36	High
F	Male	70	30	High
G	Female	69	33	High
H	Female	71	26	Intermediate
I	Female	84	14	High
J	Female	79	27	Intermediate
K	Male	71	32	High
L	Male	68	35	Intermediate
M	Female	69	27	Intermediate
N	Male	73	29	Intermediate
O	Female	71	33	Low
P	Male	83	22	Low
Q	Female	68	29	Intermediate
R	Female	89	18	Intermediate

a eHealth Literacy Scale score is used to measure digital literacy. Range 8‐40 points; a higher score indicates better perceived digital literacy [28-30].

b Level of education is divided into low, intermediate, and high in accordance with the national Dutch Statistical Office [27].

**Figure 1.**
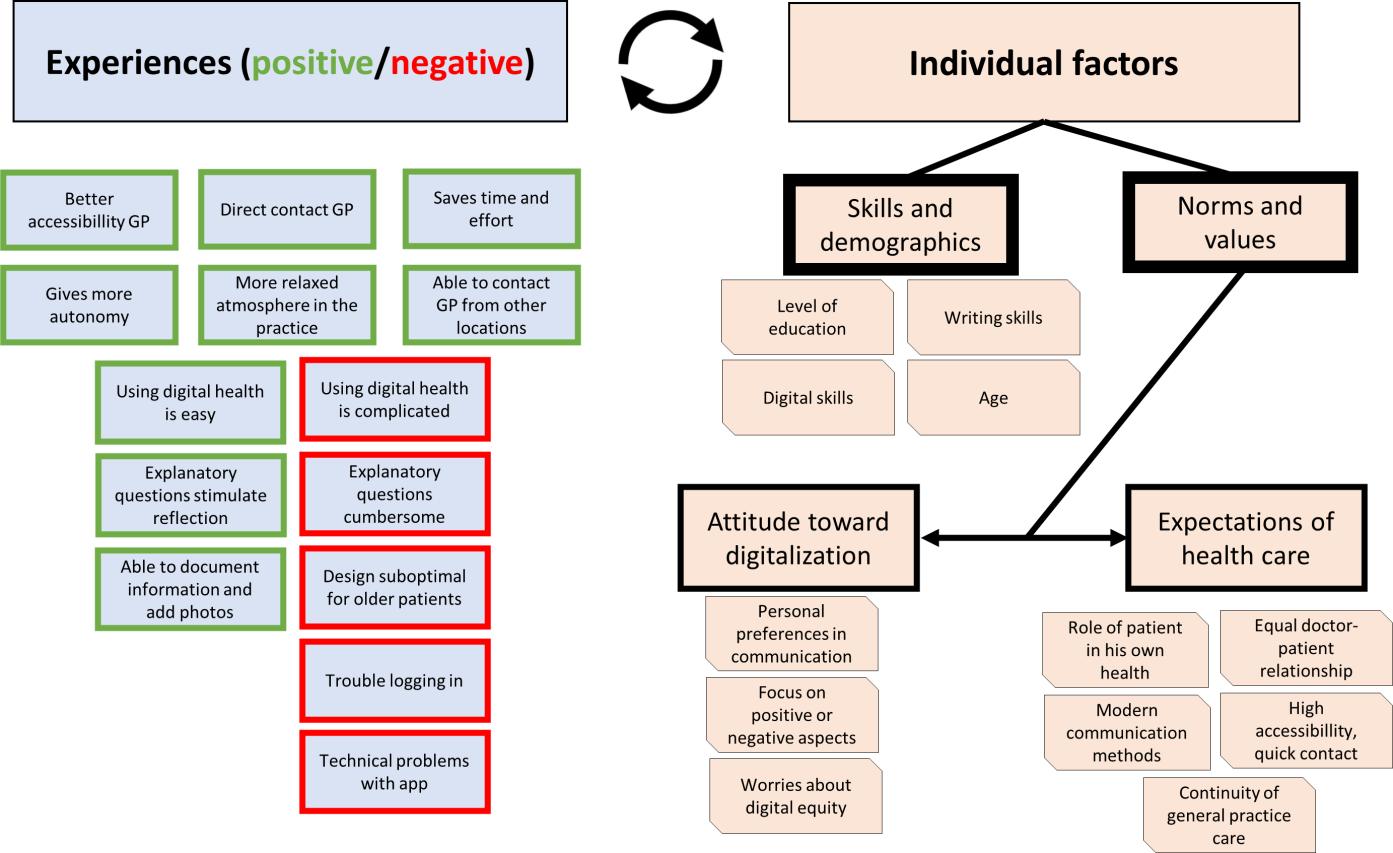
Digital health for older patients in general practice. GP: general practitioner.

We generated three main themes: (1) experiences of older people with digital health care in general practice, (2) impact of individual factors on digital health experiences, and (3) reasons for choosing a digitally oriented general practice. The first theme is divided into (1a) positive experiences with digital health care and (1b) negative experiences with digital health care. The second theme is divided into (2a) skills and demographics and (2b) norms and values. In Figure 1, we show a graphical overview of themes 1 and 2 with their subthemes.

### Theme 1: Experiences of Older People With Digital Health Care in General Practice

#### Subtheme 1a: Positive Experiences With Digital Health Care

Several participants had good experiences with the digital health platform of their general practice. They found the app easy to navigate and user-friendly. The speed with which they came into contact with a GP was one of the main advantages.

*Because, I have to say, when I send a message, they respond almost immediately.*

[Participant B]

Several participants found having more direct contact with their GP (without an intermediary) beneficial. Participants value the time and effort that they save by having digital asynchronous contact instead of calling or visiting the practice.

*I don’t always have to call, do I? So I can just ask questions and get answers. Yes, I like that very much. [Interviewer: And why do you prefer this instead of calling?] That takes too long. Then I first get an assistant, and either the GP is busy, or he is talking to someone else. That’s nice, I believe all that, but I have a question. And now I can ask him and I will get an answer. So I like that.*

[Participant L]

Other positive experiences were mainly due to opportunities that are specific for using a digital health platform to communicate with the practice, such as sending photos for dermatological issues, easily coming into contact with a GP when on holiday, and experiencing autonomy by making appointments and viewing laboratory results on the web. In addition, a few participants liked to have some time to reflect before they reply to a doctor, which is harder during a physical consultation. By chatting digitally, they have the opportunity to ask questions that would not always come to mind during a consult. A few participants liked the explanatory questions upfront because it helped them reflect on why exactly they contact the GP and activated them in their own health as well. A few participants experienced a more relaxed atmosphere in these general practices compared with other practices, due to an empty waiting room, the doctor being on time, and not constantly hearing ringing phones and assistants talking on the phone.

#### Subtheme 1b: Negative Experiences With Digital Health Care

Some participants experienced the app as complicated, and a few participants found the app too difficult to use at all. The main issue was logging in with different passwords and using a 2-factor authentication process. One of the other things that some participants experienced negatively is the automated explanatory questionnaire that they were asked to fill out at the start of each new contact with the practice. Participants found it either not relevant to their question (eg, they wanted to ask for test results and first had to answer questions like “at which moments do you experience your symptoms?”) or cumbersome because of the number of questions.

*I just find it a bit cumbersome....I’m not just going to ask nonsense.*

[Participant L]

Some minor issues also impacted participants’ experiences negatively. A few participants would prefer the app to open with a clear menu with different options instead of a direct question “how can we help you?”. In addition, a few participants experienced some technical hiccups, mainly when the practice just started. Furthermore, a few participants suggested the design of the app should be better suited to older patients, with big font size, buttons in the middle of the screen rather than in the corners, and multiple-choice options for ordering medication instead of typing. In addition, a few participants would have liked the possibility to use the digital app on a computer rather than a smartphone or tablet, so it is easier for older people to type longer messages.

### Theme 2: Impact of Individual Factors on Digital Health Experiences

#### Subtheme 2a: Skills and Demographics

Perceived digital skills seemed to play an important role in experiences with digital health. The eHEALS questionnaire can produce scores between 8 and 40, with higher scores representing higher self-perceived digital literacy [28-30]. The digital literacy of participants varied much, with eHEALS scores ranging from 14 to 40. One participant did not use a smartphone or other digital devices and therefore did not use the app at all.

*I don’t use the app myself....Then I should need to know how to make contact in the first place, therefore I need a password and, oh well... [Wife: he is a computer illiterate, and this is way too complicated for him.]*

[Participant P]

One participant, who scored 29 points relatively high on the eHEALS scale, was afraid that he could not keep up with the speed of digitalization.

*Because the speed of electronics and stuff, it goes so fast and so quick. And as long as we can follow it, through the apps and iPad and iPhone, I think it’s fine, very well. But it’s something that frightens me, that the moment may come when I can no longer keep up.*

[Participant N]

Next to digital skills, also reading and writing skills determined people’s experiences, as using the digital health platform mainly consists of reading and writing messages. None of the participants had difficulty reading, but a few had difficulties putting their symptoms into words while typing in the app.

*Well I think, you have to be able to clarify, as a patient. You have to...Yes, actually be able to clearly tell...what you have and what you want. But I think in that situation, I would call the front desk receptionist, if I really couldn’t figure it out, I would call.*

[Participant G]

Finally, we saw that few participants who were the oldest (age 79 years and older) more often reported bad experiences or did not use the app at all. In younger participants (younger than 78 years), the experiences differed, but all of them used the app (to some extent). Next to that, we observed that the people with a high level of education had predominantly more positive experiences than negative ones. In contrast, participants with a lower level of education more often had negative experiences with the app.

#### Subtheme 2b: Norms and Values

##### Attitude Toward Digitalization

Personal preferences in communication seemed to be a factor in experiences with digital health. Many participants considered chat-based communication to be of added value because it is fast, they can do it in their own time (asynchronous communication), and it is accessible. However, several participants preferred contact via telephone or face-to-face. These communication methods were more familiar to them and were viewed as more personal. In contrast, a few participants stated chat-based communication was something they would participate in with family and friends but not with a doctor. Many of the participants thought video calling was not of added value; however, some saw it as an added value for situations in which they are immobilized. Furthermore, participants’ attitude toward digitalization in society and health care appeared important. Almost half of the participants liked digitalization because it makes information more accessible and improves communication. Some people focused on disadvantages, for example, dangers in privacy and a more individualized society. Some participants simply found it redundant.

*Well I don’t think it’s necessary. It’s more, the nonsense...You are often talked into needs. You have to do this now, you have to do that now.*

[Participant D]

A few participants worried that in digital interaction, relevant emotions such as fear are lost. Opinions on the topic of searching for digital health information varied. Most participants seek information to some degree, but a few do not, mostly because digital health information can scare them. Opinions about receiving digital health information from their GP varied as well. Some liked it because it is efficient and easy, while others found it the GP’s task to inform the patient instead of sending information.

*Do I have to read what he studied for? Come on...*

[Participant J]

A factor that impacted some participants’ experiences is problems with accessibility, leading to digital inequity. To several participants, it did not impact their own experiences, but it did worry them that other older adults might not be able to access care if communication would be (solely) digital. Participants mentioned different forms of possible digital inequity. Two of those are the difficulty of digital apps (discussed in Subtheme 1b) and the level of digital skills (discussed in Subtheme 2a). Other concerns that were mentioned were that assistance with using digital health care is hard to arrange, learning and remembering new things are harder for older patients, the use of digital tools can be complicated by medical issues, and certain monitors (eg, blood pressure monitor) needed for home measurement may not be affordable or easy to use by all patients. However, some participants also mentioned that to them accessibility was increased by digital care due to easier contact, the possibility to have contact while abroad, and having insight into their own test results.

##### Expectations of Health Care

Participants’ expectations of health care in general also affected how they viewed digital health care. Some participants mentioned that they expect their GPs to apply modern communication methods.

*At the previous general practitioner, prescriptions were still sent to the pharmacy by fax. And...you couldn’t email, you couldn’t chat. You were purely like “there are so many people waiting in front of you” on a landline phone. So yeah, their way of doing things was a little outdated, if you know how things are here.*

[Participant F]

Many participants said that they expect their GP to listen carefully, take them seriously, and provide solutions. Some participants talked about valuing an equal doctor-patient relationship, in which a doctor empowers a patient by involving them in the diagnosis and decisions. They found that the app increased empowerment because when chatting, both patients and GPs use first names, the GP sends information for the patient to read themselves and involves them with different options for the next steps (eg, come to practice, wait, try lifestyle changes, and try medication). In the general practices, messages by patients were answered by the team of GPs, depending on who worked that day, to ensure each question in the app was answered as soon as possible. Some participants who valued continuity of care found it a disadvantage that the messages were not always answered by their own regular GP. However, other participants who valued accessibility and logistics more did not see this as a problem.

*[Interviewer: what is important to you in contact with your GP?] Accessibility. For this the app is fantastic because they don’t have to be open at all....As long as they respond. Right? Well, they do within 24 hours. What else do you want? Well, I think it’s great.*

[Participant D]

The way older participants viewed self-management of health varied. A few participants mentioned they did not want to play an active role in their own health management. They preferred to let the GP decide which actions were needed and preferred to have all measurements conducted in the practice. However, many participants preferred an active role in their health in some way. For instance, they were open to perform measurements at home and send them to the GP via the app. One participant stated that he saw his health as his own responsibility.

*Yes exactly! Yes, it’s my health. And I think it’s great that I can talk to a doctor about that. Not only can, but sometimes must....But it’s still my thing.*

[Participant C]

### Theme 3: Reasons for Choosing a Digitally Oriented General Practice

For many participants, the digital health platform of these practices did not play a role in their choice of practice. Most participants chose their practice because of practical reasons (location of practice, room to enlist new patients, and partner joined the practice) or because they were unsatisfied with their previous GP. Some participants chose these practices because they wanted a more modern practice (different ways of communication and young doctors) or they wanted more accessible communication. For some participants, however, the presence of digital tools was one of the reasons to enlist.

*To me it is easier to write something down if I have something, than to go to the doctor right away. So now I am more in contact with the doctor than I would have if I had to go to a normal doctor.*

[Participant M]

The reasons for choosing the digitally oriented general practices did seem to affect patients’ experiences. Participants for whom the digital tools did not play a role in their choice or who chose the practices because of different reasons (eg, the partner enlisted so they joined) were slightly more negative about the digital health platform and mainly found it complicated. In contrast, participants who enlisted in these practices because they had bad experiences with their previous GP or wanted an improvement in communication had predominantly positive experiences with the digital health platform. The same applies for participants who enlisted in these practices specifically because they wanted to use more digital health tools.

## Discussion

### Principal Results

Our main aim in this study was to investigate the experiences of older patients in general practice with a digital health platform that offers 2-way chat-based communication between the patient and GP. As expected, participants reported both positive and negative experiences with the digital app. The most important advantages of the digital health platform were described by participants as increased accessibility of the general practice for different health complaints, direct contact with the GP without an intermediary, and saving time because of asynchronous communication between the patient and GP. Disadvantages were that for some participants, use of the platform was complicated, logging in with 2-step authentication was difficult, and filling out the automated explanatory questionnaire was found to be cumbersome or irrelevant to their care request.

Our secondary aims were to assess barriers and facilitators for the use of the app and investigate if a practice focus on digital health impacted older patients’ choice to enlist. Our findings suggest that many different individual factors impacted people’s experiences with digital health care. Barriers to the use of the digital health platform in general practice appeared to be low digital and writing skills, higher age, and a low level of education. Negative attitudes toward digitalization in general, a preference for face-to-face contact with the GP, and a wish for all health questions to be handled by their own regular GP were also seen as barriers to use the app in its current form. There were also some app-related barriers in details of design and user-friendliness such as small font size. Facilitators for using the digital health platform were being digitally skilled, being highly educated, having a preference for an equal patient-GP relationship, the good accessibility of a GP, and a wish to unburden the GP. Surprisingly, for many older participants, the special focus of the practice on digital health (which was also advertised on the website) did not play a role in their choice to enlist. Logistical reasons or bad experiences with their previous GP were the main reasons to switch to their current general practice.

### Strengths and Limitations

Our study has several strengths. First, to find a representative sample of older patients who used the digital health platform, we invited all patients aged 65 years and older who were enlisted in the general practices. To find a more heterogeneous sample, we conducted a second invitation round so we were able to select more patients who were relatively old, less digitally skilled, and less educated. In the end, by using purposive sampling and continuing until the research team decided the data set was rich and complex enough, we were therefore able to attain various samples in terms of age, sex, level of education, and digital literacy. Second, all interviews were performed by the same interviewer, and the interview location was chosen by the participant. This assured that there was no interrater difference and helped participants speak freely during the interviews.

There were also some limitations. First, some participants did not use the digital app but answered how they think they would experience the functions of the digital health platform. Therefore, not all participants were able to comment on all aspects of the digital health platform. However, because expectations of experiences of older patients with digital health may help guide the development of future digital health platforms, we decided to also describe these hypothetical experiences separately. Second, our conclusions on the digital literacy of our participants should be interpreted with care. Digital literacy is a complex concept to measure. We chose to use the eHEALS because this is the only widely used questionnaire that is validated for use in Dutch older adults [28-30]. However, the eHEALS only focuses on perceived literacy as stated by patients and misses questions about skills like logging in with passwords. For future studies, we would consider complementing the eHEALS questionnaire with another established questionnaire such as the Mobile Device Proficiency Questionnaire to measure digital literacy more accurately [29,35].

### Interpretation of the Results and Comparison With Prior Work

As far as we know, this is the first study to assess the experiences of older patients with a digital health platform that offers 2-way chat-based communication in Dutch general practice. The willingness for older patients to digitally communicate with their doctor has been often described [10,15,36]. The advantages of digital health platforms mentioned by our older participants, such as increased accessibility of the general practice [37], direct contact with the GP without an intermediary [38], and time-saving because of asynchronous communication [37-39], were also found in previous studies. The problems participants in our sample experienced with logging in are considered a barrier for many digital health platforms [40]. Our participants mentioned that at a high age, it is already hard to remember passwords, while a 2-step authentication is even more complicated. However, for data security reasons, this 2-step authentication is legally obligated by the Dutch General Data Protection Regulations [41]. Although the importance of privacy of health care data cannot be overstated, current requirements limit accessibility of digital health for older patients and may therefore increase digital inequity [40]. Developing methods to ease the log-in process for end users, while still attaining adequate data security, will be essential to ensure digital health for all in the future. Swedish studies that evaluated the GP perspective of a digital health platform with 2-way chat-based communication were very positive about using automated questionnaires to better triage and prepare visits to the general practice [24,42]. This could be an important tool in reducing workload in general practice. However, in our study, some participants experienced the automated questionnaire as cumbersome or not relevant. Explaining to patients why these questions matter and attempting to keep this questionnaire as succinct and to the point as possible will be necessary to achieve broad implementation.

In our study, we found potential barriers and facilitators for the use of digital health by older patients in general practice. Because this is a qualitative study, no inferences can be made about the prevalence of phenomena, and further quantitative research will be necessary to further explore these findings [43]. However, the impact of age, digital literacy, and level of education on digital health has been often described in previous studies and implies a digital divide in which people have unequal access to important parts of society [15-17,44]. A more novel finding in our study is the way how expectations of older patients from general practice care affected their experience with digital health. We found that valuing an equal doctor-patient relationship that causes patient empowerment may be a facilitator. However, patients who wanted all health questions to be handled by one regular GP were less happy with the current form of the digital health platform. This is an important finding because personal continuity of care is a core value of general practice and offers multiple benefits to both patients and GPs [45]. When general practices use a digital health platform, choices have to be made when handling care requests. The digital health platform we assessed allowed all care requests to be primarily answered by a GP who was on duty, even when this was not the patient’s regular GP. This ensured that questions that request immediate care could also be asked digitally but limited personal continuity of general practice care. The value of personal continuity of care in general practice has been well demonstrated for both patients and GPs [46,47]. However, the optimal way to achieve personal continuity in a digital health context is still mostly unknown and deserves further study [37,48]. Early in development, future digital health platforms in general practice should view improving personal continuity of care as an essential feature. By focusing on strengthening this core value of general practice, the introduction of digital health can change from a threatening development to a protective factor. Our study showed that the reasons for older patients to enlist in a practice were mostly practical. Surprisingly, for most older participants, the option of digital health care did not influence their choice for the practice. This is at odds with previous studies that stated that digitally oriented practices attract only digitally minded, young, and skilled patients [37,38,49]. This is further confirmation that digital health platforms for general practice should be designed to be accessible to all to reduce the risk of increased health disparities by the digital divide.

### Implications for Research and Practice

#### For Practice

This research shows that a digital health platform in general practice with 2-way chat-based communication can offer benefits to older patients, but nondigital routes remain important. When developing digital health platforms, it is important to think of details that improve user-friendliness for older patients. An automated explanatory questionnaire may help make the GP work efficiently, but for patients, it should be succinct and to the point, and its importance should be explained clearly. When general practices consider implementing digital health platforms, it is good to know that for older patients this aspect may not play a role in enlisting in a practice.

#### For Research

To ensure digital health for all, future studies should focus on other patient groups who may struggle with the use of digital health platforms in general practice. Interviewing younger patients with a low socioeconomic position, low level of education, or low (digital) literacy could complement our findings. Furthermore, we found several potential barriers and facilitators for older people in using digital health. Large-scale quantitative research could be helpful to further assess the effects of these factors in daily practice. Finally, continuing scientific work on the effects of digital health platforms on the core value of personal continuity of care will be essential to ensure that digital health can be sustainably implemented in general practice.

### Conclusions

This qualitative study showed both positive and negative experiences of older patients in general practice with a digital health platform that offers 2-way chat-based communication between patients and GPs. In assessing barriers and facilitators for the use of the app, we found that individual factors impacted older patients’ experiences with digital health care such as skills, norms and values, attitudes toward digitalization, and expectations of general practice care. For many older participants, the digital profile of the general practice did not play a role in their choice to enlist. Further improvement of digital health platforms will be necessary to ensure digital health for all in general practice.

## Supplementary material

10.2196/59168 Multimedia Appendix 1 Automated explanatory questionnaire.

10.2196/59168 Multimedia Appendix 2 Topic list.

10.2196/59168 Checklist 1 COREQ (Consolidated Criteria for Recording Qualitative Research) checklist.

## Abbreviations

COREQ: Consolidated Criteria for Recording Qualitative Research
eHEALS: eHealth Literacy Scale
GP: general practitioner
