# Supplementary material for: Understanding Older Adults’ Experiences With a Digital Health Platform in General Practice: Qualitative Interview Study
Source: JMIR Aging. 2024 Aug 30;7:e59168. doi: 10.2196/59168 (PMC11378695; doi:10.2196/59168)
Supplement: Multimedia Appendix 1 [file aging-v7-e59168-s001.docx]

Starting question when opening the app: How can we help you?

Follow-up questions:

1. How severe are your symptoms?
2. At which moments do you experience your symptoms?
3. Since when do you have your symptoms?
4. How did your symptoms start?
5. Do you have any idea about what the diagnosis might be?
6. How do you feel about these symptoms?
7. Did you already do something to decrease the symptoms?
8. Would you like to explain the symptoms in more detail?

Then people can choose a couple of options:

1. Discuss symptoms with the general practice
2. Make an appointment (either face to face or video)
3. Save the answers to the questions but don’t send them yet

If they choose one of the first two options, their answers to the questions are sent to the practice and they get two automatic replies:

1. Thank you for clarifying your concern/symptoms.
2. What is your question to the general practice?
