# Supplementary material for: Understanding Older Adults’ Experiences With a Digital Health Platform in General Practice: Qualitative Interview Study
Source: JMIR Aging. 2024 Aug 30;7:e59168. doi: 10.2196/59168 (PMC11378695; doi:10.2196/59168)
Supplement: Multimedia Appendix 2 [file aging-v7-e59168-s002.docx]

| Subject | Sample question |
| --- | --- |
| Questions about the respondents’ background | What is your age? |
|  | How many years have you attended school? (from kindergarten to any further education) What kind of school(s) did you attend? |
|  | What kind of work have you done? |
|  | What have been your experiences with digital devices in your work? |
|  | What are your experiences with digital devices in private life? What kind of devices do you use? What do you use them for? (e.g. for communication, internet, ordering things online, search routes, banking) |
|  | How do you view the digital developments in society? |
| Questions about Doccs general practice | What were your reasons for registering at Doccs general practice? |
|  | Did their digital way of work play a role in your choice? |
|  | What are your experiences being a patient in this practice?  Do your experiences match your expectations? |
| Contact with your GP | What is important to you in contact with your GP (when you are in the consulting room)? |
|  | What is important to you in contact with your GP or with the general practice before you are there? (e.g. for making a appointment, for asking small questions) |
|  | What is in your opinion the role of digital tools in communication with your GP and/or general practice? |
| Use of the Doccs app | Do you use the Doccs app? |
|  | What are your experiences with the Doccs app? |
|  | Are you satisfied? |
|  | Are there things that don’t work for you? Are there things you would like to be improved? |
|  | What would make it easier for you to use the app / start using the app? |
|  | Does it have added value to you to be able to chat with the GP?  Does it have added value to you to be able to video call with the GP? |
| Digital health | Disregarding of this app of Doccs and how it works, if everything is possible, what would you like to use digital health apps for in contact with your GP?  (e.g. making appointments, asking questions, repeating medication, tracking health data like glucose levels or bloodpressure and making them realtime visible for your GP, getting lifestyle advices and being able to track that and send to the GP, …) |
| Health information | Do you search the internet yourself for information about your health? |
|  | How do you feel about receiving trustworthy online medical information (thuisarts.nl) from the GP in which you have to search for the answer to your question yourself? |
|  | How do you feel about receiving trustworthy online medical information (thuisarts.nl) from the GP after your consult, so you can use it to look things up you might have forgotten. |
|  | How do you feel about doing more about your health yourself, for example by measuring your blood pressure at home and sending the results to the GP? |
