# Supplementary material for: Understanding Older Adults’ Experiences With a Digital Health Platform in General Practice: Qualitative Interview Study
Source: JMIR Aging. 2024 Aug 30;7:e59168. doi: 10.2196/59168 (PMC11378695; doi:10.2196/59168)
Supplement: Checklist 1 [file aging-v7-e59168-s003.docx]

| Domain 1: Research team and reflexivity | |  |
| --- | --- | --- |
| Personal Characteristics | |  |
| 1 Interviewer/facilitator | Which author/s conducted the interview or focus group? | Hanna Knotnerus |
| 2 Credentials | What were the researcher's credentials? | BSc Medicine, currently last year MSc Medicine |
| 3 Occupation | What was their occupation at the time of the study? | Full time master student medicine, research assistant |
| 4 Gender | Was the researcher male or female? | Female |
| 5 Experience and training | What experience or training did the researcher have? | She was trained to perform semi-structured interviews by dr. Vincent van Vugt. He is experienced by his GP specialty training and research during his PhD. |
| Relationship with participants | |  |
| 6 Relationship established | Was a relationship established prior to study commencement? | No. The participants were called after they responded that they wanted to participate. In the call HK talked through the informed consent (which they already received on paper at home). |
| 7 Participant knowledge of the interviewer | What did the participants know about the researcher? e*.g. personal goals, reasons for doing the research* | The participants were informed about HK’s study in medicine and the stage she was in. |
| 8 Interviewer characteristics | What characteristics were reported about the interviewer/facilitator? e.g. *Bias, assumptions, reasons and interests in the research topic* | The participants were informed about HK’s study in medicine and the stage she was in. They were also informed that HK was not linked to the Doccs general practices and therefor had no interest in specific answers. |
| Domain 2: study design | | |
| Theoretical framework | | |
| 9. Methodological orientation and Theory | What methodological orientation was stated to underpin the study? | We used a reflexive thematic analysis approach according to Braun and Clarke.[32-34] After reading the transcripts several times the data was coded by two authors (HK, HN) with an inductive orientation. At the time, HK was a medicine master student in her final year of training, and HN was a general practice resident and PhD student. HK had an open view towards digital health for older adults in general practice, but was mindful of dangers it may pose to accessibility of care. Especially for older patients. HN shared those concerns, but as a PhD candidate who researched the implementation of an online treatment for chronic dizziness in general practice, she also had a lot of positive experiences with older patients employing digital health. HK and HN separately coded the first two transcripts and then reflected together on the story within the data. After their discussion, HK continued coding the other transcripts to further analyze the data. Next, HK assigned codes to generate initial themes and subthemes which were visualized in a mindmap. This preliminary analysis was discussed in a meeting with HK, HN and VvV. Afterwards HK continued analyzing the data, also performing selective coding in which she conceptualized each theme further, searched for relations across cases and analyzed variation within and between the cases. She visualized this in mindmaps, code matrices and code-relation matrices to gain insight into the spectrum of different factors influencing patients’ experiences. The final results were discussed with the project team (HK, VvV and OM). Interviews were analysed in Dutch using MAXQDA (version 2022). After completion, all themes, subthemes, codes and quotes were translated into English by the authors. |
| Participant selection |  |  |
| 10 Sampling | How were participants selected? | We used purposive sampling by phone to create as much heterogeneity in the respondents group in age, gender, level of education, digital skills and experiences with the Doccs app. |
| 11 Method of approach | How were participants approached? | Physical mail send to their home address, containing two letters:   1. Invitation letter from Doccs 2. Information letter from the Amsterdam UMC about the study and their privacy   People could respond by handing in a contact form at the general practice, sending an e-mail or sending the contact form physically using the retour envelope. |
| 12 Sample size | How many participants were in the study? | 18. |
| 13 Non-participation | How many people refused to participate or dropped out? Reasons? | 36 respondents were not interviewed. Mainly because they had characteristics or experiences that were already represented in the group with interviewed respondents. |
| Setting |  |  |
| 14. Setting of data collection | Where was the data collected? | Patients’ home, general practice and 1 interview at the Amsterdam University Medical Center (location AMC). |
| 15. Presence of non-participants | Was anyone else present besides the participants and researchers? | No. |
| 16. Description of sample | What are the important characteristics of the sample? | See Table 1. |
| Data collection |  |  |
| 17. Interview guide | Were questions, prompts, guides provided by the authors? | Yes we had a topic list for the interviews, see appendix C. |
| 18. Repeat interviews | Were repeat interviews carried out? If yes, how many? | No. |
| 19 Audio/visual recording | Did the research use audio or visual recording to collect the data? | Yes, all interviews were audio-recorded using the secured Philips Voice Recorder app. |
| 20 Field notes | Were field notes made during and/or after the interview or focus group? | Brief notes were made during the interviews. |
| 21 Duration | What was the duration of the interviews or focus group? | 22 to 66 minutes per interview. |
| 22 Data saturation | Was data saturation discussed? | Yes. HK, HN, VvV and OM discussed richness and complexity of data during the interviewing and analysis process to decide the number of interviews. |
| 23 Transcripts returned | Were transcripts returned to participants for comment and/or correction? | No. |
| Domain 3: analysis and findings | | |
| Data analysis |  |  |
| 24 Number of data coders | How many data coders coded the data? | Two, HK and HN. |
| 25 Description of the coding tree | Did authors provide a description of the coding tree? | Yes. |
| 26 Derivation of themes | Were themes identified in advance or derived from the data? | The themes were derived from the data. |
| 27 Software | What software, if applicable, was used to manage the data? | We used MAXQDA (version 2022). |
| 28 Participant checking | Did participants provide feedback on the findings? | No. |
| Reporting |  |  |
| 29 Quotations presented | Were participant quotations presented to illustrate the themes / findings? Was each quotation identified? | Yes, we used multiple quotations in the article, each identified with a participant number. |
| 30 Data and findings consistent | Was there consistency between the data presented and the findings? | Yes. |
| 31 Clarity of major themes | Were major themes clearly presented in the findings? | Yes, there were three major themes generated: (1) experiences of people; (2) the influence individual factors on people’s experiences; and (3) reasons to choose for a digitally oriented general practice. |
| 32 Clarity of minor themes | Is there a description of diverse cases or discussion of minor themes? | Yes, within each theme diverse cases were discussed. |
